# Cooperative effects of matrix metalloproteinase and cyclooxygenase-2 inhibition on intestinal adenoma reduction

**DOI:** 10.1038/sj.bjc.6600867

**Published:** 2003-04-29

**Authors:** R A Wagenaar-Miller, G Hanley, R Shattuck-Brandt, R N DuBois, R L Bell, L M Matrisian, D W Morgan

**Affiliations:** 1 Department of Cancer Biology, Vanderbilt University, Nashville TN 37232, USA; 2 Division of Animal Care, Vanderbilt University, Nashville TN 37232, USA; 3 Abbott Laboratories, Abbott Park, IL 60064-3500, USA; 4 TAP Pharmaceutical Products Inc., Lake Forest, IL 60045, USA; 5 Department of Medicine, Vanderbilt University, Nashville TN 37232, USA

**Keywords:** matrix metalloproteinase, cyclooxygenase-2, multiple intestinal neoplasia, colon cancer

## Abstract

Matrix metalloproteinases (MMPs) and cyclooxygenase-2 (COX-2) are expressed in both sporadic and familial adenomatous colonic polyps and tumours and have been independently shown to play causal roles in intestinal tumour formation in mouse models of colon cancer. The apparent roles of these enzymes in intestinal tumorigenesis led us to examine, in the *Min* mouse model of colon cancer, if selective COX-2 and MMP inhibitors provide additive or synergistic therapeutic benefits in intestinal tumour prevention. The broad-spectrum MMP inhibitor (A-177430; MMPI) and the selective COX-2 inhibitor (A-285969; COX-2I) both showed dose-dependent inhibition of the number of adenomas in *Min* mice. Using suboptimal doses, the MMPI reduced tumour multiplicity by 32%, the COX-2I by 48% and, both agents in combination resulted in a 67% decrease compared to control demonstrating a cooperative effect on intestinal tumorigenesis. Apoptosis, proliferation, and angiogenesis were assayed in tumors from each treatment group. These agents in combination allowed for a lowered dosage to be administered to achieve significant biological effects. Clinically, this could potentially reduce side effects associated with currently used MMP and COX-2 inhibitors. Together, these compounds could represent an easily tolerated chemopreventive approach.

Colon cancer is the third largest cause of cancer-related deaths in both men and women in the US. Sporadic colorectal cancer is a multistep process involving a number of genetic and epigenetic changes (Fearon and Vogelstein, 1990; Kinzler and Vogelstein, 1996), while familial forms such as familial adenomatous polyposis (FAP) and hereditary nonpolyposis colorectal cancer (HNPCC) have been shown to be caused by mutations in the tumour suppressor adenomatous polyposis coli (APC) and mismatch repair (MMR) genes, respectively. There are multiple stages and many years involved in the progression from benign colon polyp to invasive carcinoma and metastatic disease. In addition to efforts to improve therapy for colorectal cancer, the detection of early-stage lesions by colonoscopy provides the opportunity to develop novel chemopreventive approaches to this disease.

Inhibitors of cyclooxygenase (COX, prostaglandin H synthase), the enzyme that catalyses the conversion of arachidonic acid to prostaglandin, have been utilised as chemopreventive agents for colorectal cancer. There are two forms of the COX enzyme; COX-1 is expressed ubiquitously while COX-2 expression is induced in response to a number of different stimuli including cytokines, growth factors, oncogenes, serum, and tumour promoters (Kujubu *et al*, 1991; O'Banion *et al*, 1991). COX-2 is expressed in 85–90% of human colorectal adenocarcinomas (Eberhart *et al*, 1994; Kargman *et al*, 1995; Sano *et al*, 1995). Initial studies of COX and colorectal tumours came about after epidemiological studies linked nonsteroidal anti-inflammatory drug (NSAID) intake with reduced risk for developing colorectal tumours (Thun *et al*, 2002). Clinical trials in FAP patients with NSAIDs showed that their use results in significant reduction in both the number and size of the polyps (Hawk *et al*, 1999). These studies also demonstrated that NSAIDs had adverse side effects of bleeding and ulceration, which was most likely because of inhibition of gastric prostaglandin production by COX-1.

A number of animal models have been used to determine the effectiveness of COX inhibition in the treatment and prevention of colorectal cancer. Among these is the multiple intestinal neoplasia (*Min*) mouse (Williams *et al*, 1996), which carries an ethylnitrosourea-induced germline mutation at codon 850 in exon 15 of the APC gene that results in a truncated protein (Su *et al*, 1992). Within the context of the C57Bl/6 genetic background, 100% of mice with the *Min* mutation develop adenomas throughout the intestinal tract (Moser *et al*, 1990). NSAIDs such as piroxicam, indomethacin, and sulindac were all effective in preventing intestinal tumorigenesis in this model (Beazer-Barclay *et al*, 1996; Gann *et al*, 1993; Marnett, 1995; Reddy, 1987; Wargovich *et al*, 1995; Jacoby *et al*, 1996). Furthermore, mice null for COX-2 in an APC mutant background (APCΔ716) develop 86% fewer tumours than mice with COX-2 expression (Oshima *et al*, 1996).

In addition to altered expression of COX-2, members of the matrix metalloproteinase (MMP) family are induced in colon cancer (McDonnell *et al*, 1991). The MMPs are a growing family of enzymes, currently consisting of more than 20 mammalian family members, which have been shown to play a role in tumours originating in several different organs (for review see Nelson *et al*, 2000). MMPs are zinc-dependent enzymes capable of degrading extracellular matrix components. A number of different MMPs may be important in intestinal tumour formation as human tumours have been shown to express interstitial collagenase (MMP-1), gelatinase A (MMP-2), stromelysin 1 (MMP-3), matrilysin (MMP-7), gelatinase B (MMP-9), and stromelysin 3 (MMP-11) (Newell *et al*, 1994; Nelson *et al*, 2000). MMP-7, an epithelial specific MMP, is expressed focally in 50% of benign colonic adenomas and overexpressed in 85% of human malignant colorectal tumours (Newell *et al*, 1994). Additionally, MMP-7 is overexpressed in 90% of adenomas from patients with FAP (Takeuchi *et al*, 1997).

The *Min* mouse model of gastrointestinal tumorigenesis has also been used to indicate that MMPs play causative roles in tumour formation. We have previously shown that 70 – 90% of *Min* adenomas express MMP-7 in the neoplastic epithelium (Wilson *et al*, 1997; Shattuck-Brandt *et al*, 1999) and that *Min* mice null for MMP-7 have 58% fewer tumours than *Min* mice with wild-type MMP-7 (Wilson *et al*, 1997). Tumour multiplicity in *Min* mice treated with a broad-spectrum MMP inhibitor, batimistat (BB94), was decreased by 48% as compared to vehicle-treated mice (Goss *et al*, 1998). In addition to MMP-7 expression, 60–65% of *Min* adenomas express MMP-2 and MMP-10 (stromelysin-2) and 50% express MMP-3 and MMP-13 (collagenase) with expression limited to the stroma surrounding the adenoma (Wilson *et al*, 1997).

The similarity in tissue expression of both MMP-7 and COX-2 and the apparent roles that these enzymes have in intestinal tumorigenesis suggested that their expression may be coregulated in polyps of the *Min* mouse. Although both enzymes were expressed at high levels in the *Min* adenomas, *in situ* and immunohistochemistry localised MMP-7 specifically to the neoplastic epithelial cells, while COX-2 was localised to the superficial stroma within the adenoma (Shattuck-Brandt *et al*, 1999). Recently, COX-2 has been shown to be expressed in the polyp-associated fibroblasts and endothelial cells of intestinal polyps from *Apc*Δ716 mice (Sonoshita *et al*, 2002). Additionally, a high percentage of human colorectal tumours expressed both MMP-7 and COX-2, although, the levels and localisation of MMP-7 and COX-2 expression within the tumours did not correlate (Shattuck-Brandt *et al*, 1999). It is also important to note that neither COX-2 nor MMP-7 is expressed in normal intestinal epithelium but instead is upregulated in the tumors making inhibition of both of these enzymes an ideal therapeutic target. These results suggested that MMP-7 and COX-2 are not coregulated and that there is no direct relation between the expression of COX-2 and the expression of MMP-7.

Since COX-2 and MMP-7 are not coregulated and are unlikely to be part of the same signal-transduction pathway, we hypothesised that combination therapy with selective COX-2 and MMP inhibitors may provide additive or synergistic therapeutic benefits in the *Min* mouse model. We report that the administration of a broad-spectrum MMP inhibitor (A-177430) and a selective COX-2 inhibitor (A-285969) to *Min* mice significantly suppresses intestinal tumorigenesis, and that administration of both the MMP inhibitor (MMPI) and the COX-2 inhibitor (COX-2I) in combination further reduces intestinal tumorigenesis to a level that is greater than either compound individually.

## MATERIALS AND METHODS

### Animals

C57Bl/6-*Min* (*Min*) mice (Jackson Laboratory, Bar Harbor, ME, USA) were maintained as previously described with littermates housed under identical conditions in microisolator cages (Wilson *et al*, 1997). Pups were weaned at 3 weeks of age and maintained *ad libitum* on diet 5015 (Purina Mills Inc. LabDiet, Richmond IN, USA). Analysis of the *Min* allele was performed as described using a PCR assay (Whitehead and Joseph, 1994). Mice were weighed twice weekly and gained weight at normal rate. All animal protocols were approved by Vanderbilt University Animal Care and Use Committee.

### Therapeutic agents

**Figure 1:**
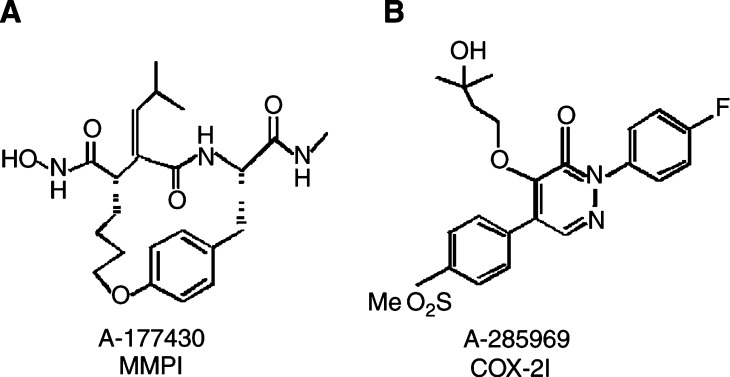
Structure of MMPI A-177430 (**A**) and COX-2I A-285969 (**B**).

A-177430 and A-285969 were synthesised at Abbott Laboratories (Abbott Park, IL, USA; Figure 1

). Single-agent study: mice were administered A-177430 at a dose of 100 mg kg^−1^ (200 mg kg^−1^ day^−1^, *n*=6) suspended in 0.2% hydroxypropylmethylcellulose (HPMC), A-285969 at 40 mg kg^−1^ (80 mg kg^−1^ day^−1^, *n*=5) suspended in HPMC. Sulindac was given as a positive control for tumour inhibition and was given at a dose of 1.875 mg kg^−1^ (3.75 mg kg^−1^ day^−1^, *n*=6) while HPMC-injected mice were used as control (*n*=3). Combination study: mice were administered A-177430 at a dose of 30 mg kg^−1^ (60 mg kg^−1^ day^−1^, *n*=6) suspended in 0.2% HPMC, A-285969 at 20 mg kg^−1^ (40 mg kg^−1^ day^−1^, *n*=5) suspended in HPMC, both A-177430 and A-285969 at the doses used in single compound treatment (*n*=6), or vehicle (HPMC) alone (*n*=5). All compounds were administered by intraperitoneal (i.p.) injection twice daily for 45 days beginning at 5 weeks of age. The twice daily dose of 40 and 20 mg kg^−1^ of A-285969 was chosen because pilot studies have shown A-285969 to be active in a mouse cornea model of angiogenesis at 50 mg kg^−1^ (Leal J and Bell R, unpublished data) and because doses of 20 mg kg^−1^ have shown to be safe on the gastrointestinal tract of dogs (Bell R in preparation). The twice daily dose of 100 and 30 mg kg^−1^ of A-177430 was chosen because these doses have been previously shown to be effective in inhibiting tumour growth of the MatLyLu prostate cancer cell line in rats (Rabbani *et al*, 2000). Mouse pharmacokinetics give a half-life of less than 4 h for both the MMPI and COX-2I indicating the need for twice daily dosing. No clinical data are available regarding possible toxicities of either of these compounds in humans.

### Analysis of tumour formation

For analysis of intestinal tumours, animals were killed by CO_2_ asphyxiation. Intestinal tissue was fixed overnight in 4% paraformaldehyde (Fluka Chemika-Biochemica, Switzerland) in 1 × PBS and transferred to 70% ethanol for storage at 4°C. Intestines were cut longitudinally and tumours were analysed under a dissecting microscope at × 10 magnification. Tumour sample identifications were masked and the number, diameter, and distribution of tumours were recorded.

### Immunohistochemistry

Tumours were dissected out of the fixed intestines and embedded in paraffin. Sections (5 *μ*m) were cut and mounted on Superfrost Plus slides (Fisher Scientific, Pittsburgh, PA, USA). Slides were stained for apoptosis using the ApopTag Kit (Intergen, Purchase, NY, USA) according to the manufacturer's instructions. Nuclei were visualised by counterstaining with Contrast Green. Apoptosis results were quantified by counting the number of positively stained tumour-associated nuclei per 1000 total tumour-associated nuclei. Results are expressed as percent positively stained nuclei.

Analysis of cell proliferation was performed with a monoclonal mouse anti-Ki67 antibody (Novocastra Laboratories Ltd., Newcastle upon Tyne, UK). Antigen retrieval was performed with Target Retrieval solution (DAKO Laboratories, Carpinteria, CA, USA) according to the manufacturer's instructions. Negative controls consisted of sections that were incubated in control IgG antibody. Endogenous peroxidase and biotin were quenched with 0.03% hydrogen peroxide and Biotin Blocking System (DAKO Laboratories, Carpinteria, CA, USA), respectively. A primary antisera dilution of 1 : 50 was prepared using the Animal Research Kit/Peroxidase (DAKO Laboratories, Carpinteria, CA, USA) biotinylation and blocking reagents and slides were incubated for 6 h. Diaminobenzidine (DAB) plus was used to produce localised, visible staining followed by a haematoxylin counterstain.

Immunohistochemistry results were quantified by counting the number of positive tumour-associated nuclei out of the total number of tumour-associated nuclei with a minimum of 600 nuclei analysed for each tumour. Results are expressed as percent of nuclei staining positive.

Analysis of angiogenesis was performed with a rabbit anti-human Von Willebrand factor (VWF) antibody obtained from DAKO Laboratories. Endogenous peroxidases were quenched with 0.6% hydrogen peroxide. Antigen retrieval was accomplished by proteinase K digestion in a solution of 4% proteinase K for 30 min at room temperature. Sections were incubated in control IgG antibody (negative control) or rabbit anti-VWF antibody (1 : 2000) overnight at 4°C and developed with the Vectastain ABC kit from Vector Laboratories (Burlingame, CA, USA) according to the manufacturer's instructions. Immunohistochemistry staining for VWF was quantified using Zeiss Image (Carl Zeiss, Inc., Thornwood, NY, USA) and results are expressed as percent of total tumour area stained.

### Statistics

All values were determined using the StatView program (SAS Institute Inc, Cary, NC, USA). A value was determined to be statistically significant if the *P*-value is less than 0.05 as determined with the nonparametric Mann–Whitney test.

## RESULTS

### Effect of therapeutic agents on tumour multiplicity

The MMP inhibitor A-177430 (referred to as MMPI) is a previously described broad-spectrum MMP inhibitor (Sheppard *et al*, 1998), which contains a hydroxamic acid moiety that chelates the MMP active site zinc ion and inhibits enzymatic activity with IC_50_ values of 2–6 nM for MMPs -1, -2, -3, -7, and -9. This MMPI has been used successfully at 100 mg kg^−1^ to inhibit tumour growth and metastasis in rats inoculated with MatLyLu rat prostate cancer cells (Rabbani *et al*, 2000). A-285969 (referred to as COX-2I) is a selective COX-2 inhibitor that shows 60-fold selectivity against COX-2 *vs* COX-1 in human whole-blood assays. It also potently inhibits COX-2-driven prostaglandin formation in rodents (oral ED_50_ 0.7 mg kg^−1^), prostaglandin-driven inflammation (ED_50_=1.0 mg kg^−1^) in adjuvant arthritis in rats, and angiogenesis in a mouse cornea model of angiogenesis (50 mg kg^−1^) (R Bell, in preparation).

**Figure 2:**
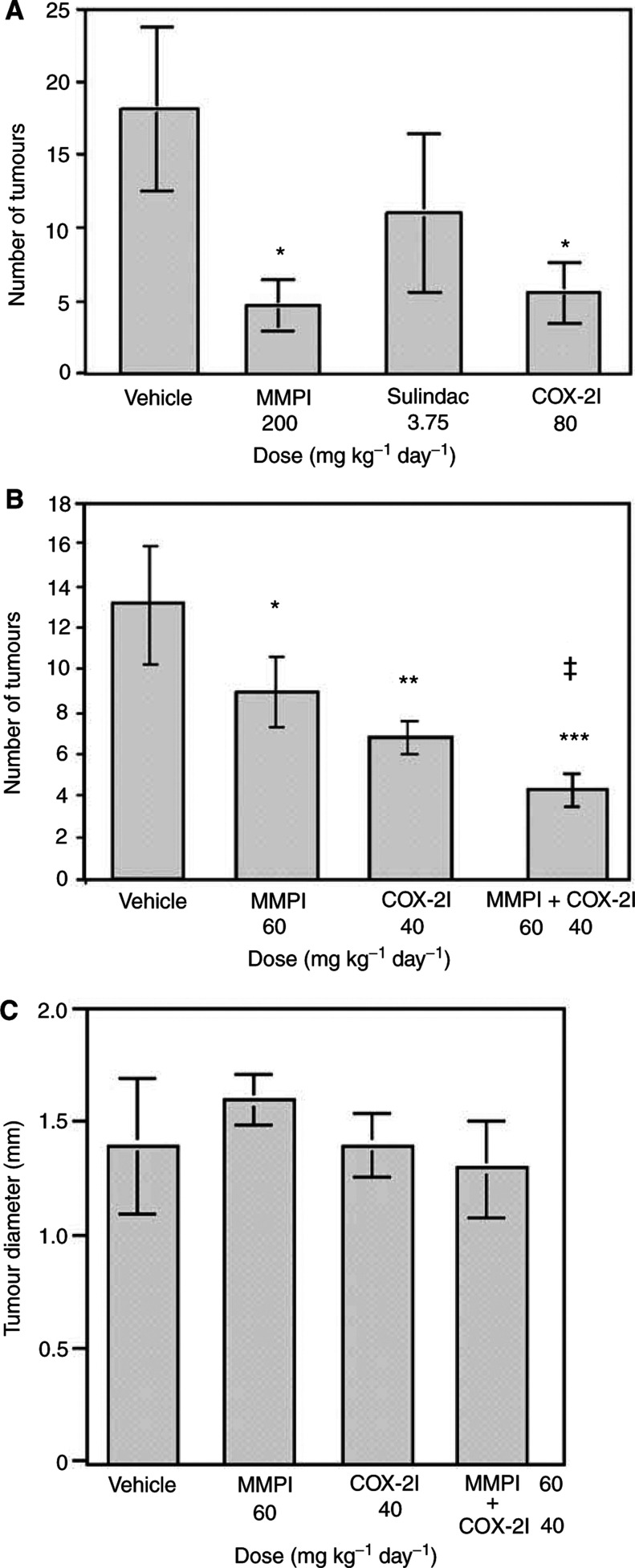
MMPI (A-177430) and COX-2I (A-285969) cooperate to reduce tumour multiplicity in *Min* mice. Single-dose study (**A**) involved treatment of Min mice at 5–6 weeks for 45 days with twice daily i.p. injections of vehicle (HPMC, *n*=3), a broad-spectrum MMPI (A-177430, *n*=6), a COX-2I (A-285969, *n*=5), or an NSAID (sulindac, *n*=6) at the doses indicated. ^*^Denotes statistically significant difference between individual treatment groups and the vehicle-treated group. Combination study (**B**) repeated the above regimen using suboptimal doses alone and in combination as indicated. Each bar is representative of the total number of mice in the experiment where vehicle *n*=5, COX-2I *n*=5, MMPI *n*=6, and combination *n*=6. Statistical differences were calculated as described in Materials and Methods by comparing vehicle and treated groups ^*^P=0.0367, ^**^P=0.009, ^***^P=0.0062 or the combination-treated group and individual treatment groups. ^‡^*P*=0.0062. Tumour size (**C**) was determined as described in Materials and Methods.

The ability of these compounds to alter tumour multiplicity was examined using 5 to 6-week-old *Min* mice. In the single-agent study, mice were treated for 45 days with vehicle (HPMC), MMPI at 100 mg kg^−1^ (200 mg kg^−1^ day^−1^) or COX-2I at 40 mg kg^−1^ (80 mg kg^−1^ day^−1^) as indicated in Materials and Methods. The NSAID sulindac was used as a positive control. Both the MMPI and the COX-2I reduced tumour multiplicity by 69% (5.6±2.5) and 64% (6.4±1.5), respectively, compared to vehicle-treated controls (18±2). Sulindac was less effective than either of the MMPI or the COX-2I in this experiment (Figure 2A

).

To determine if there is a cooperative effect of inhibiting both the MMPs and COX-2 pathways, the experiment was repeated using lower doses of the MMPI and COX-2I separately and in combination. Lower doses of the two compounds were used than in the single-agent study to ensure if the two inhibitors cooperated, additive or synergistic effects could be observed. *Min* mice were treated with vehicle (HPMC) or suboptimal doses of MMPI at 30 mg kg^−1^ (60 mg kg^−1^ day^−1^), COX-2I at 20 mg kg^−1^ (40 mg kg^−1^ day^−1^) or both MMPI and COX-2I at the same doses used in the individual treatments as indicated in Materials and Methods. Tumour multiplicity was reduced in MMPI-treated mice by 32% (9.0±1.7; *P*=0.0367) and by 48% (6.8±0.84; *P*=0.0090) in COX-2I-treated mice as compared to vehicle-treated mice (13.2±2.8) as shown in Figure 2B. Treatment with a combination of MMPI and COX-2I decreased tumour multiplicity by 67% (4.3±0.82; *P*=0.0062) as compared with vehicle-treated mice. Additionally, the reduction in tumour number in combination-treated mice was significantly greater than mice treated with either compound individually (Figure 2B). These results most closely represent an additive effect for these drug interactions as determined by the algebraic method (Berenbaum, 1977). The average tumour diameter was not affected by treatment with any of the therapeutic agents (Figure 2C). No weight loss or alterations in feeding were noted in mice treated with either of the doses of MMPI or COX-2I or the combination of the two compounds (data not shown). No gastrointestinal ulcers were noted by gross analysis of intestines from any of the treatment animals.

### Effect of therapeutic agents on tumour apoptosis

**Figure 3:**
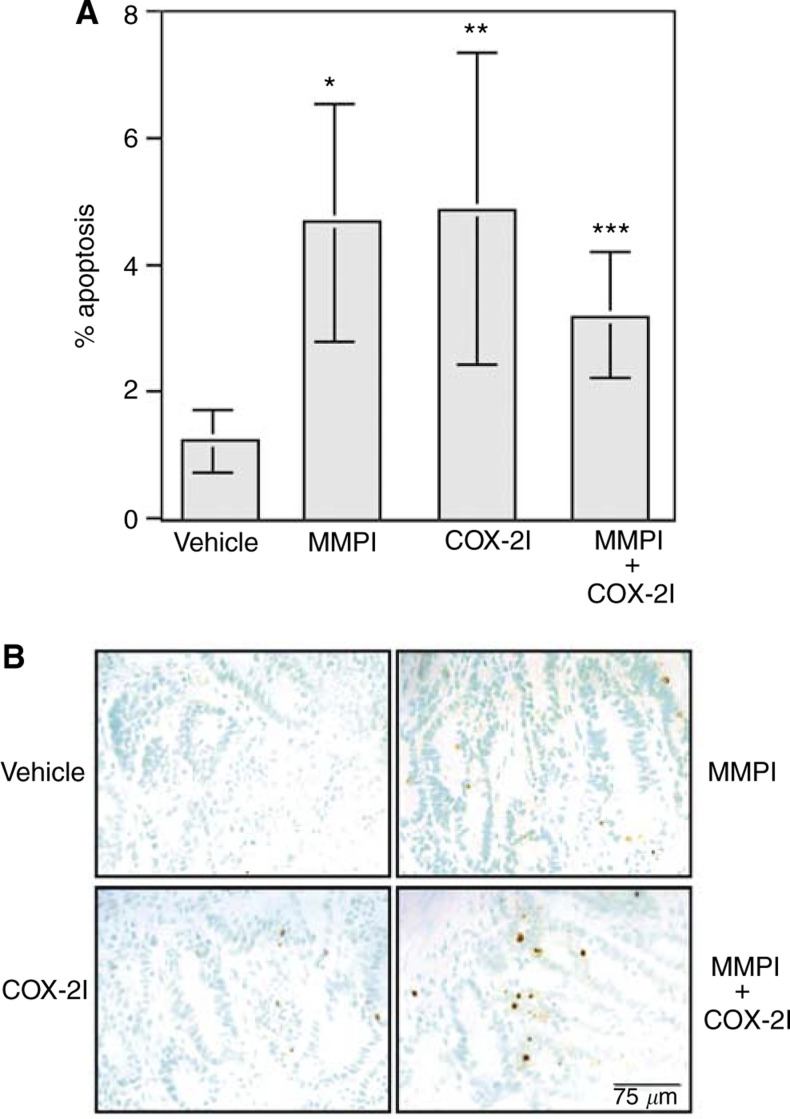
Tumours from Min mice treated with MMPI or COX-2I have increased levels of apoptosis in comparison with vehicle-treated mice. Intestinal tumour sections were stained for apoptosis using the ApopTag kit and number of positive nuclei and total tumour-associated nuclei were counted as described in Materials and Methods. (**A**) A total of 1000 nuclei were counted per tumour. Results are represented as percent nuclei staining positive. Each bar is representative of total number of tumours scored for each group where vehicle *n*=11, MMPI *n*=10, COX-2I *n*=10, and combination *n*=6. Statistical differences were calculated as described in Materials and Methods by comparing vehicle and treated groups. ^*^P=0.0012, ^**^P=0.0018, ^***^P=0.009. (**B**) Representative staining for apoptosis from each treatment group. Apoptotic nuclei are stained brown and contrast green is used to visualize total nuclei.

Intestinal tumour sections were assessed for DNA fragmentation to determine if an increased rate of apoptosis may be a mechanism by which MMPI and COX-2I decreased *Min* tumour multiplicity. Tumours from mice treated with MMPI had a 3.9-fold increased apoptotic index compared with tumours from vehicle-treated mice, with 1.25±0.50% apoptotic nuclei in tumours from vehicle-treated mice to 4.83±2.87% apoptotic nuclei in tumours from MMPI-treated mice (Figure 3A

; *P*=0.0012). Similarly, tumours from mice treated with COX-2I exhibited an increased apoptotic index of four-fold as compared with vehicle-treated mice (1.25±0.50% apoptotic nuclei in vehicle mice to 4.91±2.47% apoptotic nuclei in mice treated with COX-2I, Figure 3A; *P*=0.0018). Additionally, tumours from mice treated with the combination of the MMP and COX-2 inhibitors had a 2.5-fold increase in the apoptotic index (3.22±0.97%; *P*=0.0090) compared to tumours from mice treated with vehicle; however, there was no significant increase in the rate of apoptosis over treatment with either MMPI or COX-2I alone (Figure 3A). Representative staining is shown in Figure 3B.

### Effect of therapeutic agents on tumour proliferation

**Figure 4:**
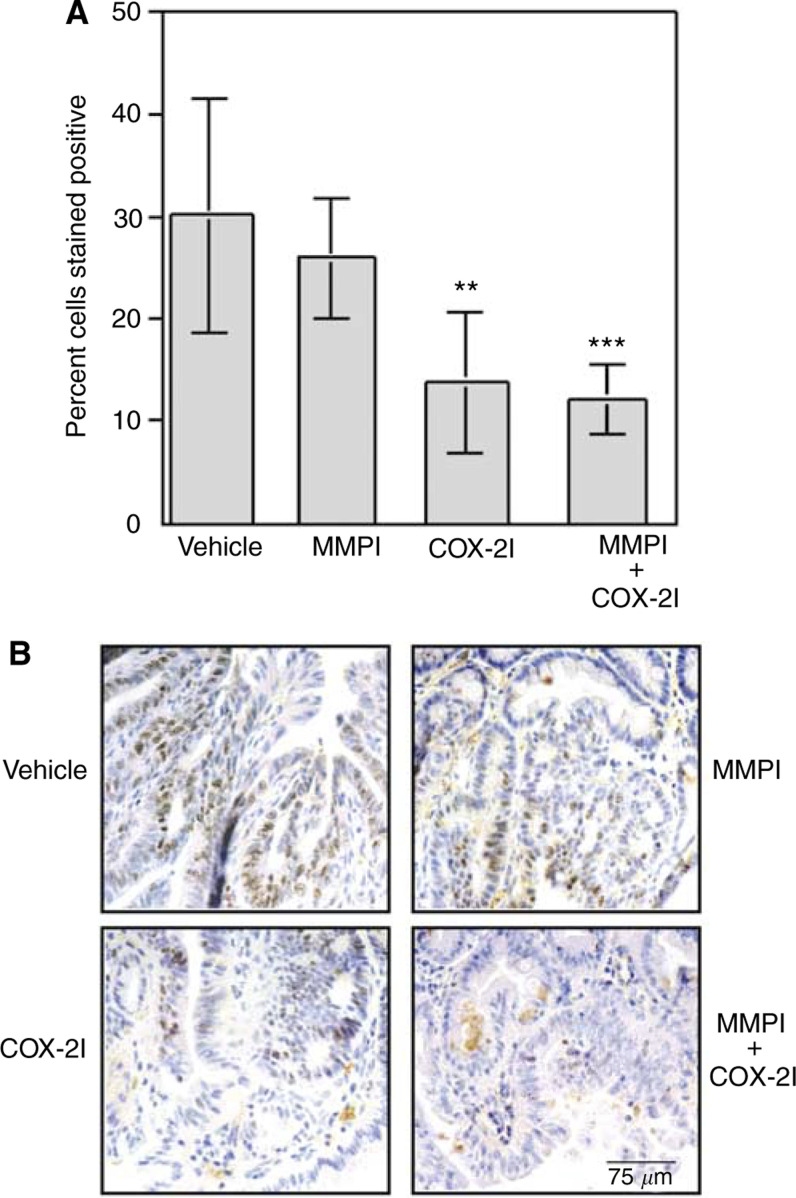
Cell proliferation is significantly inhibited in tumours from *Min* mice treated with COX-2I but not with MMPI. Immunohistochemistry was performed with a Novocastra anti-human Ki67 antibody as described in Materials and Methods. (**A**) A minimum of 600 total tumour-associated nuclei were counted and results are represented as percent of nuclei staining positive out of total tumour-associated nuclei. Each bar is representative of total number of tumours scored for each group where vehicle *n*=7, COX-2I *n*=5, MMPI *n*=13, and combination *n*=5. Statistical differences were calculated as described in Materials and Methods by comparing vehicle and treated groups. ^**^P=0.0185, ^***^P=0.0058. (**B**) Representative examples of Ki67 staining for each treatment group. Proliferative nuclei are stained brown and all nuclei are counterstained blue with hematoxylin.

Tumour sections were stained for Ki67 to determine if MMPI and COX-2I alone or in combination altered the level of cell proliferation. Ki67 is a nuclear antigen expressed in proliferating cells during late G1, S, M, and G2 stages of the cell cycle. *Min* tumours treated with MMPI did not exhibit a statistically significant decrease in cell proliferation compared to tumours from control-treated animals (25.9±5.8% and 30.1±11.4%, respectively, Figure 4A

). Tumours from *Min* mice treated with COX-2I, however, exhibited significantly reduced levels of cell proliferation from 30.1±11.4%, in vehicle-treated mice to 13.8±6.9% (Figure 4A; *P*=0.0251). Tumours from Min mice treated with the combination MMPI and COX-2I did not exhibit a significantly different rate of proliferation than tumours from mice treated with the COX-2I alone (Figure 4A). Representative staining is shown in Figure 4B.

### Effect of therapeutic agents on angiogenesis

**Figure 5:**
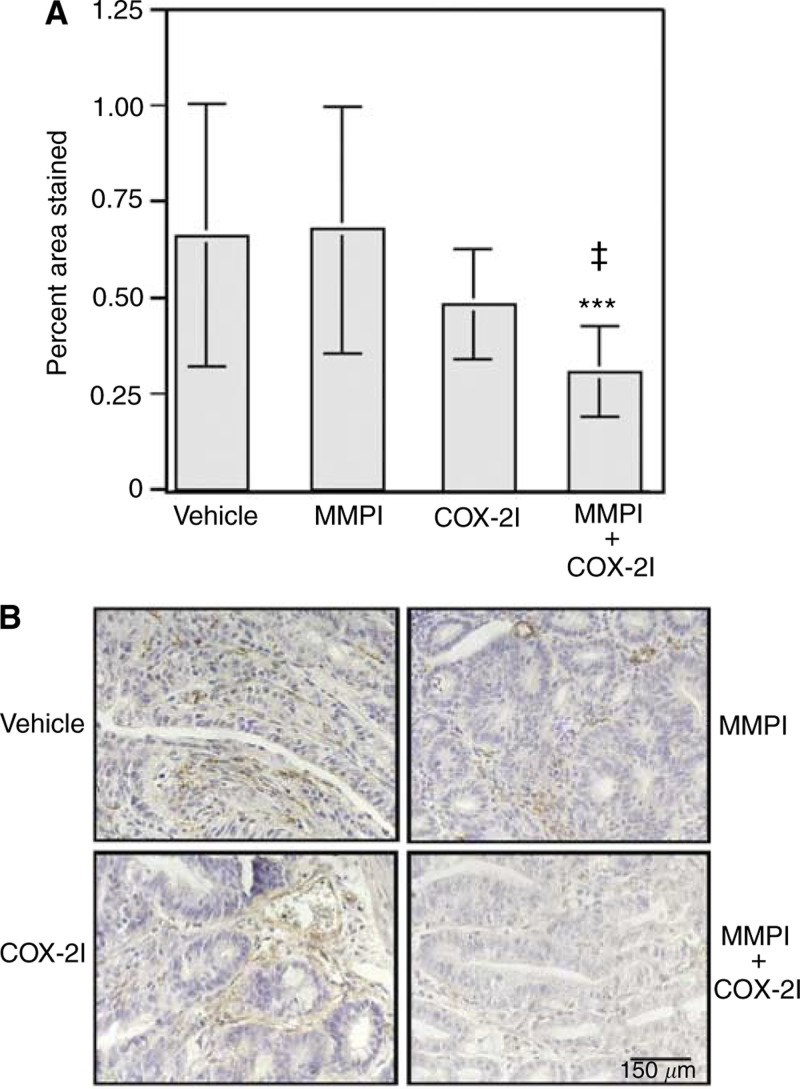
Combination treatment with both MMPI and COX-2I inhibits angiogenesis in *Min* intestinal tumours, while single-agent therapy does not alter angiogenesis. Immunohistochemistry for Von Willibrand Factor (VWF) was performed with a DAKO anti-VWF antibody as described in Materials and Methods. (**A**) Quantification of immunostaining was performed using Zeiss Image. Each bar is representative of the total number of tumours scored for each group where vehicle *n*=9, MMPI *n*=8, COX-2I *n*=6, and combination *n*=6. Statistical differences were calculated as described in Materials and Methods by comparing vehicle-and combination-treated group. ^***^P=0.0005, ^‡^MMPI and combination *P*=0.0009 and COX-2I and combination *P*=0.0031. (**B**) Representative examples of VWF immunohistochemistry for each treatment group. VWF-positive vessels are stained brown and all nuclei are counterstained blue with haematoxylin.

Intestinal tumour sections from each treatment group were stained for VWF to determine if MMPI or COX-2I used alone or in combination could alter angiogenesis. Staining was quantified using Zeiss Image and is presented as percent of total tumour area. No significant difference in staining for VWF was observed in tumours from mice treated with MMPI or COX-2I alone (Figure 5A

). However, treatment with MMPI and COX-2I in combination significantly reduced the amount of VWF staining when compared to vehicle (*P*=0.0005) or MMPI (*P*=0.0009) and COX-2I (*P*=0.0031) used individually (Figure 5A). Representative staining is shown in Figure 5B.

## DISCUSSION

The purpose of this study was to determine if the combination of a broad spectrum MMP inhibitor (A-177430) and a selective COX-2 inhibitor (A-285969) could cooperate to reduce intestinal tumour formation in *Min* mice. We have previously shown that MMPs and COX-2 are expressed in a high percentage of *Min* tumours (Wilson *et al*, 1997; Shattuck-Brandt *et al*, 1999). It has also been shown that elimination of MMPs or COX-2 by either genetic strategies or with inhibitors results in a decrease in the number of tumours formed in mice with altered APC function (Wilson *et al*, 1997; Goss *et al*, 1998). We hypothesised that treatment of intestinal cancer with both MMP inhibitors and COX-2 inhibitors could result in a further decrease in tumour number and/or size as compared to treatment with the individual inhibitors.

Both the MMPI and the COX-2I decreased *Min* tumour multiplicity in a dose-dependent manner. These results are consistent with previously published data using structurally distinct MMP and COX-2 inhibitors (Beazer-Barclay *et al*, 1996; Jacoby *et al*, 1996; Oshima *et al*, 1996). These data provide further support to the contention that these enzymes contribute to intestinal tumour progression and represent valid chemotherapeutic targets for colon cancer. No effect on tumour diameter was observed in any of the treatment groups at any of the doses tested. These data are consistent with previously published data in which MMPs have been inhibited using a structurally distinct MMPI and with tissue inhibitors of metalloproteinases (TIMPs), endogenous inhibitors of MMPs (Goss *et al*, 1998). It is unclear if longer treatment may have resulted in observable differences in tumour size. The novel aspect of these studies is the observation that combination treatment of the *Min* mice with both MMP and COX-2 inhibitors resulted in an additive decrease in the average tumour number.

The mechanism of inhibition of tumour number was investigated by assaying for tumour apoptosis, proliferation, and angiogenesis. The MMPI alone raised the level of tumour cell apoptosis, but did not significantly affect proliferation or angiogenesis. An increase in apoptosis is apparently sufficient to reduce the number of detectable adenomas. The apoptotic activity of MMPs and of their endogenous inhibitors, TIMPs, is a controversial subject. In some cases, MMPs have been shown to be proapoptotic while in other cases they are implicated to be antiapoptotic (for review see Egeblad and Werb, 2002; Jiang *et al*, 2002). These contradictory results may be partially explained by the different cell and tissue systems under examination. In our study, it is clear that chronic administration of an MMP inhibitor results in increased apoptosis in intestinal adenomas supporting a role for MMPs in the survival of benign intestinal epithelial cells.

The COX-2I also increased tumour cell apoptosis, consistent with previous reports of proapoptotic effects of COX-2 inhibition (Ballif *et al*, 1996; Chan *et al*, 1998; Hsu *et al*, 2000). The combination of the MMPI and the COX-2I had no additional statistically significant effect on the apoptotic index. This raises the possibility that these compounds alter converging pathways that affect tumour cell apoptosis. COX-2 has been shown to increase the levels of MMP-2, -9, and -14, while NSAIDS and COX-2 inhibitors have been reported to alter the levels of MMP-1, -2, -3, -9 (Ito *et al*, 1995; Takahashi *et al*, 1997, 1999; Attiga *et al*, 2000; McLaren *et al*, 2000; Callejas *et al*, 2001; Jiang *et al*, 2001). Recent reports have shown that NSAIDs inhibit MMP expression and activation through both COX-2-dependent and independent mechanisms such as through modulation of gene transcription via the ERK/Sp1 signalling pathway or through induction of the membrane-anchored MMP inhibitor RECK (Pan *et al*, 2001; Liu *et al*, 2002; Pan and Hung, 2002). Thus, the COX-2 effect on apoptosis may be mediated by alterations in MMP activity, explaining why there is no additional proapoptotic benefit of inhibiting both pathways.

The COX-2I reduced adenoma proliferation as determined by Ki67 staining. Several studies have shown prostaglandins to be important modulators of cell proliferation (Sheng *et al*, 1997; Castano *et al*, 2000). More recently, expression of COX-2 has been shown to be cell cycle dependent in human fibroblasts (Gilroy *et al*, 2001) and associated with G1 delay in intestinal epithelial cells (DuBois *et al*, 1996). Even earlier than cell growth, COX-2 inhibitors block carcinogenesis in animal models (Prescott and Fitzpatrick, 2000), and recently the Hla laboratory has shown that overexpression of COX-2 in mice was sufficient to cause tumorigenicity (Liu *et al*, 2001). Older nonselective agents and more recently new selective COX-2 inhibitors have been shown to have anticancer effects in animal models and some human tumours (Fosslien, 2000). Thus, the proapoptotic and antiproliferative effects of COX-2 inhibition presented here are consistent with observations in other models.

The most interesting observation of these studies is the effect of the MMPI and COX-2I on tumour-associated angiogenesis. Neither compound significantly altered angiogenesis alone at the doses given, but the combination resulted in a 53% reduction in VWF-stained blood vessels. Previous studies have indicated that both COX-2 and MMP inhibition have antiangiogenic effects (Jones *et al*, 1999; Stetler-Stevenson, 1999; Williams *et al*, 2000). Thus, it is possible that higher doses of either compound would have shown significant alteration in VWF-stained blood vessels, implying that a critical threshold must be reached to observe inhibition of angiogenesis. The combination of low doses of both agents may have acted in an additive fashion and been sufficient to cross this threshold. Alternatively, the MMPI and COX-2I may have acted on different pathways in a synergistic manner to affect angiogenesis. Additional dose–response studies would be required to distinguish between these two possibilities. It is unclear if the target in this case is the tumour epithelium or endothelium. Enzymes produced in tumour epithelium may be involved in stimulating pathways that release angiogenic factors. Alternatively, MMPs or COX-2 produced by endothelial cells may be essential for their growth, viability, or ability to make new blood vessels. Both MMPs and COX-2 are expressed in tumour-associated endothelial cells and inhibition of these enzymes could stimulate the cells to undergo apoptosis. The COX-2 inhibitor Celecoxib has been previously been shown to induce apoptosis in endothelial cells (Leahy *et al*, 2002). TUNEL analysis of tumour sections show that the majority of the apoptosis occurs in the epithelial component of the tumour; however, some TUNEL-positive cells can be seen within the stromal component and may be endothelial cells (Figure 2). In preliminary studies, we have observed a cooperativity between low doses of the MMPI and COX-2I in inducing apoptosis in cultured endothelial cells (data not shown). Thus, the target of the inhibitors may be both tumour epithelium and endothelium.

MMP inhibitors have been successfully used in preclinical models to demonstrate that MMPs are important in several different tumour types (Nelson *et al*, 2000). In general, the results of Phase III clinical trials in advanced stage cancers have been disappointing, although the broad-spectrum MMPI marimastat showed some efficacy in patients with gastric cancer (Fielding *et al*, 2000). Several reasons for the clinical failure of MMPIs have been proposed, including the inappropriate testing in advanced-stage disease settings (Coussens *et al*, 2002). The ability to detect premalignant disease and the progressive nature of colorectal cancer makes it amenable to treatment at early stages, and provides a model for testing of novel preventive strategies. MMPIs, like COX-2Is, are ideal candidates for chemoprevention agents in trials with patients with recurring polyps (Marnett and DuBois, 2002).

Treatment with two different inhibitors with independent signalling pathways may decrease the toxicity of drugs by reducing the dose necessary to achieve the same effect. Recent findings (Torrance *et al*, 2000) demonstrate that combination treatment of *Min* mice with NSAIDs and EGF receptor kinase inhibitors cooperate to inhibit tumour multiplicity. Additionally, the combination of an MMP inhibitor with a COX-2 inhibitor may be used with traditional cytotoxic agents, based on promising results observed with such agents used in combination with either a MMP or COX-2 inhibitor (Neri *et al*, 1998; Fosslien, 2000). These data together with our data suggest that a combination of agents may be effective in the prevention of colon cancer. These findings are significant in that they represent potential chemopreventative targets for treatment or may be a more easily tolerated chemotherapeutic treatment for colon cancer.
